# L'endométriose périnéale profonde sur cicatrice d’épisiotomie: à propos d'un cas rare

**DOI:** 10.11604/pamj.2013.16.112.3415

**Published:** 2013-11-23

**Authors:** Meriem Laadioui, Fdili Alaoui, Sofia Jayi, Hakima Bouguern, Hikmat Chaara, Moulay Aabdelilah Melhouf

**Affiliations:** 1 Université sidi mohammed benabdellah, CHU Hassan II de Fes, Maroc

**Keywords:** endométriose, épisiotomie, diagnostic, traitement, pronostic, endometriosis, episiotomy, diagnosis, treatment, prognosis

## Abstract

Parmi les localisations rares de l'endométriose sur cicatrice, celle du périnée demeure exceptionnelle, l'origine en est souvent iatrogène (épisiotomie). Nous rapportons le cas d'une patiente présentant une douleur cyclique, au niveau de la cicatrice d’épisiotomie. Avec à l'examen clinique une masse de 3,5 cm de grand diamètre au niveau de la cicatrice d’épisiotomie. L’écho périnéale a objectivé une image hypoéchgène hétérogène non vascularisée en regard de la cicatrice d’épisiotomie faisant 3,23/1cm. L'excision de la lésion a été réalisée et l’étude anatomopathologique a confirmé le diagnostic d'endométriose. Les suites postopératoires étaient simples avec un recul de 3 mois sans récidive de la douleur ni de la masse. A travers notre cas et une revue de la littérature, nous insistons sur les la nécessité du diagnostic clinique et d'une prise en charge précoce en vue d'améliorer le pronostic de cette entité rare.

## Introduction

L'endométriose périnéale correspond à la présence de tissu endométrial au niveau du périnée superficiel. Des antécédents à type d’épisiotomie, de déchirure obstétricale ou d'intervention périanale associée souvent à un curetage sont fréquemment retrouvés. L'endométriose anopérinéale est l'objet d'une faible littérature regroupant quelques articles sporadiques souvent d'un seul cas clinique. La première observation d'endométriose périnéale a été rapportée en 1923 et celle d'endométriose du canal anal en 1968. Nous rapportons un nouveau cas d'endométriose localisée à la cicatrice d’épisiotomie, à travers lequel et à la lumière d'une revue de la littérature nous insistons sur toutes les caractéristiques de cette entité notamment les moyens diagnostiques, la prise en charge thérapeutique et le pronostic de cette pathologie rare.

## Patient et observation

**Figure 1:**
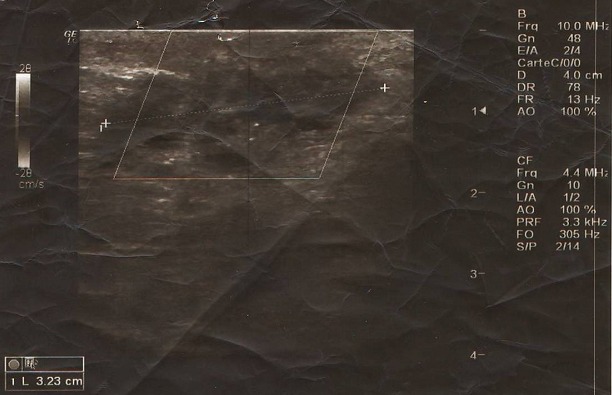
Aspect échografique du nodule endométriosique

**Figure 2:**
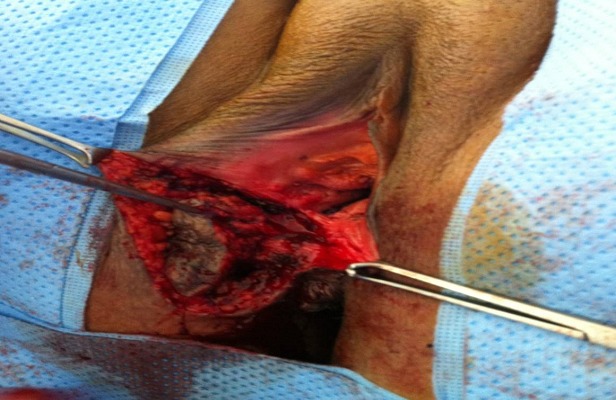
Incision vulvaire montrant le nodule endométriosique

**Figure 3:**
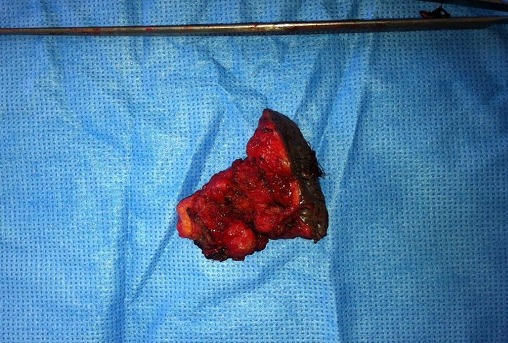
Pièce opératoire: lésions fibreuses avec présence de tissu endométrial à l'examen anatomo-pathologique

**Figure 4:**
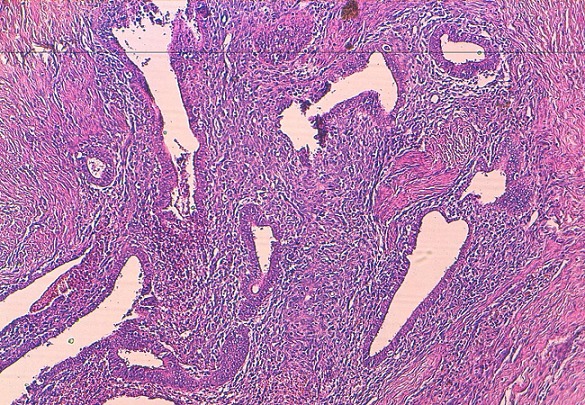
Aspect histologique du nodule endométriosique au moyen grossissement, coloration standard HES

Mme M. M. patiente âgée de 34 ans, troisième geste deuxième pare, ayant comme antécédent une fausse couche spontanée non curetée, un enfant né par césarienne âgé de 13 ans et un enfant né par voie basse avec épisiotomie âgé de 5 ans. Ses cycles sont réguliers sous dispositif intra-utérin. Elle consulte pour douleurs pelviennes cycliques au niveau de la cicatrice d’épisiotomie remontant à 1 an. L'examen clinique trouve une masse de 3,5 cm au niveau de la cicatrice d’épisiotomie. L’échographie périnéale a objectivé une image hypoéchogène hétérogène non vascularisée en regard de la cicatrice d’épisiotomie faisant 3,23/1cm (Figure 1). La patiente a bénéficié d'une résection de la masse, réalisée sous rachianesthésie, nous avons réalisé une incision biconcave autour de la cicatrice d’épisiotomie avec un décollement sous cutané emportant le nodule endométriosique arrivant jusqu’à la fosse pararéctale (Figure 2, Figure 3). L'examen anatomo pathologique a confirmé le diagnostic d'endométriose (Figure 4). Les suites postopératoires étaient simples avec une bonne évolution et un recul de 8 mois sans récidive de la masse ni de la douleur

## Discussion

L'endométriose périnéale est une entité rare. Liang rapporte une série de 6 cas [1]; Robert Akbari rapporte deux cas [2]. Cette pathologie est l'apanage de la femme en période d'activité génitale, ses signes révélateurs sont variables.

L’éthiopathogénie de cette localisation n'est pas claire, La plupart des auteurs sont pour l'hypothèse d'une implantation possible des cellules endométriales viables dans la plaie; qui peuvent se diviser, constituer des foyers d'endométriose et répondre aux stimulations hormonales cycliques [1].

Le diagnostic est à évoquer devant tout syndrome douloureux et/ou tumoral anopérinéal chez une femme en période d'activité génitale [3]. Les antécédents d'épisiotomies et les déchirures périnéales d'origine obstétricale sont souvent retrouvés. L'examen clinique trouve généralement une tuméfaction périnéale d'aspect bleutée, sensible, de volume variable selon le cycle menstruel. Son contenu d'aspect brun noirâtre est très évocateur [1], les douleurs anopérinéales sont souvent rythmées par les cycles génitaux [4]. Ainsi, l'examen périnéal doit être systématique chez toute femme présentant des douleurs pelviennes chroniques d'autant plus qu'elle a eu une épisiotomie, cet examen montre généralement le nodule périnéal et doit rechercher systématiquement des localisations anorectales et de la cloison rectovaginale. Le diagnostic différentiel de l'endométriose périnéale se pose avec l'abcès anopérinéal, dans sa forme très localisée, fluctuante, récidivante. Le mélanome anal, très rare, de couleur souvent bleutée peut être également confuse avec un nodule endométriosique [3, 4].

L'imagerie de l'endométriose est basée essentiellement sur deux examens: l’échographie et l'imagerie par résonance magnétique (IRM). En échographie, Elle prend l'aspect non spécifique de nodules habituellement hypoéchogènes et hétérogènes (selon leur composante solide et/ou liquide), parfois hyperéchogènes (formes hémorragiques), à limites externes volontiers floues et irrégulières, ayant une forme et une taille variables (selon la quantité de sang et de fibrose, le moment du cycle et/ou le traitement médical en cours) [2]. Certains auteurs ont proposé l’échographie endo-anale ou l’écho endoscopie dans les localisations profondes [2]. Le scanner n'est généralement pas indiqué. L'IRM est actuellement la meilleure méthode d'imagerie pour évaluer une endométriose. Elle trouve ses indications aussi bien dans les localisations endopelviennes que dans les localisations extra-pelviennes [5] L'efficacité de cette technique a particulièrement été démontrée dans la détection des localisations profondes [7]. L'IRM présente en plus l'avantage de pouvoir localiser d'autres implants endométriosiques à distance. Les lésions d'endométriose sur cicatrice d’épisiotomie se caractérisent en IRM par un épaississement fibreux en hyposignal franc sur les séquences pondérées T2 L'aspect est plus évocateur lorsqu'il s'agit d'une infiltration de forme stellaire et rétractile [6].

L'abstention thérapeutique est de règle dans les formes asymptomatiques (30% des endométrioses). Le traitement médical est soit prescrit seul: est efficace sur les douleurs et consiste en un arrêt des menstruations par des progestatifs (Danazol) ou analogues de la GnGH; soit en complément d'un traitement chirurgical limite ou incomplet par l'importance de la résection musculaire à effectuer. Le traitement de choix est la résection chirurgicale à adapter dans ses modalités selon l'âge des patientes [8]. Elle peut être réalisée sous anesthésie locale [9], locorégionale ou générale [8], selon la taille du nodule.

## Conclusion

Nous insistons sur l'intérêt d’évoquer le diagnostic d'endométriose périnéale chaque fois qu'une patiente présente des douleurs cycliques dans les suites proches ou lointaines d'une épisiotomie. L'examen clinique éventuellement complété par l’échographie et mieux une IRM peut renforcer le diagnostic et ceci devrait systématiquement conduire à une exérèse large ce qui permet de garantir une évolution favorable dans la majorité des cas.
